# Fertility preservation in patients with *BRCA* mutation

**DOI:** 10.3332/ecancer.2020.1033

**Published:** 2020-05-06

**Authors:** Suleiman Ghunaim, Ghina Ghazeeri, Dalia Khalife, Hatem A Azim

**Affiliations:** 1 Department of Obstetrics and Gynecology, Division of Reproductive Endocrinology and Infertility, Haifa Idriss-ART Unit, American University of Beirut Medical Center, PO Box 11-0236, Riad El Solh, Beirut 1107 2020, Lebanon; 2 Breast Cancer Centre, Hospital Zambrano Hellion, Tecnologico de Monterrey, San Pedro Garza Garcia, Mexico

**Keywords:** oncofertility, counselling, ovarian stimulation, breast cancer

## Abstract

Evidence suggests a likely negative impact of deleterious *BRCA* mutations on female fertility. Hence, different studies have aimed to address the reproductive potential and performance of fertility preservation strategies in *BRCA*-mutated breast cancer patients with a prime focus on their safety and efficacy. However, several uncertainties exist in many domains of this field. The aim of the current paper is to overview the reproductive potential and fertility preservation options in breast and ovarian cancer patients harbouring a *BRCA* mutation. We also discuss pre-implantation genetic testing in an attempt to help physicians during oncofertility counselling of these patients.

## Introduction

Nowadays, the prevalence of cancer diagnosis occurring during the reproductive age is increasing [1] with some studies showing up to three out of four female cancer patients between 18 and 45 years desiring future fertility [2]. Thus, it is imperative to address such issues as early as possible following cancer diagnosis [3].

Breast cancer is the most commonly diagnosed malignancy in premenopausal women [4]. Approximately, 5%–7% of women with breast cancer are diagnosed before the age of 40 years in the Western world. It also accounts for more than 40% of all cancers in women, with an individual average risk of 1 in 173 by the age of 40 and approximately 1 in 1,500 by the age of 30 [5].

*BRCA*1 is a tumour suppressor gene, involved in double strand DNA break repairs. It is essential in oocyte survival to help resist potential genetic stress [6] thus, any mutation leads to accelerated loss of oocyte reserve. Interesting to note, similar results are found with the *BRCA*2 carriers, however, since there is a delayed decline of the normal *BRCA*2 allele function at the end of the reproductive life, the deleterious effects are noted at a later stage [7].

In *BRCA*-mutation carriers, breast cancer often occurs during the reproductive age. Up to 12% of the cases diagnosed in young patients are hereditary tumours related to germline mutations in the breast cancer susceptibility genes *BRCA*1 or *BRCA*2. The cumulative breast cancer risk at 40 years of age is approximately 24% for *BRCA*1 carriers and 13% for *BRCA*2 carriers [8]. Not only does it increase the chance of cancer, but also it has been also suggested to negatively impact the reproductive potential of these women.

According to both the European Society for Medical Oncology [9] and American Society of Clinical Oncology guidelines [10], it is clearly recommended that healthcare providers initiate the discussion on the possibility of infertility with patients with cancer treated during their reproductive years or with their parents/guardians as early as possible. Providers should be prepared to discuss fertility preservation options and/or to refer all potential patients to appropriate reproductive specialists. Although patients may be focused initially on their cancer diagnosis, providers should advise patients regarding potential threats to fertility in the treatment process so as to allow for the widest array of options for fertility preservation.

To date, little is known regarding fertility counselling in cancer patients with *BRCA* mutation. In this review, we will discuss the role of *BRCA* mutation in ovarian function and how to counsel young cancer patients harbouring a *BRCA* mutation inquiring into fertility preservation.

## *BRCA* mutation and ovarian function

The *BRCA* genes seem to be implicated in gametogenesis. As mostly demonstrated in animal models, mice harbouring *BRCA* mutations may have a lower ovarian reserve and their oocytes have a higher frequency of impaired DNA double-strand break repair mechanism. This can be associated with decreased ability to counteract genotoxic stress leading to an accelerated loss of ovarian reserve following the accumulation of these DNA breaks in the oocyte [7].

Many studies looked at ovarian function parameters in patients harbouring *BRCA* mutation (see Table 1). A recent retrospective study looked at 795 patients subdivided into a cancer cohort and elective freezing cohorts. 176 patients were coming for fertility preservation in the cancer cohort while others presented for elective egg freezing. *BRCA* carriers were significantly younger, (32.4 ± 3.6 years versus 35.5 ± 4.3 years, *p* < 0.001) and had a longer stimulation period (*p* < 0.001 for both). There were no obvious differences between *BRCA*1 and *BRCA*2 carriers in terms of markers of ovarian reserve or stimulation response [11]. In another prospective study, patients with *BRCA*1 mutation showed higher premature ovarian insufficiency, and thus lower number of matured oocytes for oocyte cryopreservation compared to patients with *BRCA*2 [12]. Additionally, the role of *BRCA* has been shown to play a role in ovarian aging as *BRCA* mutation has been linked to an acceleration in the DNA double strand break in oocytes [13].

## Controlled ovarian stimulation in cancer patients harbouring a BRCA mutation

### Breast cancer

Embryo/oocyte cryopreservation is considered as the standard of care for fertility preservation in breast cancer patients [9, 10].

In a prospective, non-randomised, controlled study aiming to investigate the safety of ovarian stimulation for fertility preservation with letrozole supplementation prior to breast cancer treatment, patients who underwent embryo cryopreservation received an antagonist protocol for controlled ovarian stimulation, including letrozole to maintain oestrogen levels within physiological ranges during stimulation [14]. Of 337 breast cancer patients, 120 underwent the above protocol, while 217 did not undergo any fertility-preserving procedure and thus served as their controls. After a mean follow-up of 5 years, the study showed no difference in recurrence-free survival between the two groups (hazard ratio (HR), 0.77; 95% confidence intervals (CI), 0.28–2.13; *p* = 0.61). Of the whole cohort, 188 patients underwent *BRCA* mutation testing resulting in 47 *BRCA*-mutated cases. Among those, 26 underwent controlled ovarian stimulation. There was no significant difference in recurrence-free survival among the two groups, with one recurrence in the COS group and two in the control group (*p* = 0.57) [15]. Women in the *BRCA*-positive cohort tended to retrieve (6.5 versus 9; *p* = 0.145), and to cryopreserve (3.5 versus 6; *p* = 0.121) less oocytes than those in the *BRCA*-negative cohort. Poor response rate (i.e., retrieval of ≤ four oocytes) was 40.0% and 11.1% in the *BRCA*-positive and *BRCA*-negative cohorts, respectively (*p* = 0.147).

In a recent prospective study on the use of letrozole and its effect on controlled ovarian stimulation cycles, Turan *et al* [16] investigated a cohort of 145 young patients undergoing controlled ovarian stimulation for fertility preservation stimulated with an antagonist protocol either using letrozole combined with recombinant follicle-stimulating hormone (*n* = 118) or FSH alone (*n* = 24). The mean number of total (15.6 (7.9) versus 10.2 (7.8); *p* = 0.004) and mature oocytes (10.4 (5.1) versus 7.8 (3.5); *p* = 0.044) and embryos frozen (7.7 (5.3) versus 5.3 (2.7); *p* = 0.043) were significantly higher after letrozole addition to stimulation. Yet, women with *BRCA* mutations produced significantly fewer oocytes (11.0 (8.0) versus 16.4 (7.7), *p* = 0.015) and embryos (5.1 (4.4) versus 8.2 (4.7), *p* = 0.013), compared to the patients not harbouring a mutation even with the utilisation of letrozole. Letrozole seemed to be an independent factor for a higher number of total oocytes (95% confidence interval (CI): 1.9 to 3.6; *p* = 0.002) mature oocyte (95% CI: 0.3 to 1.4; *p* = 0.028) and embryo yield (95% CI: 0.7 to 1.4; *p* = 0.015) after adjusting for age, body mass index, baseline FSH level and *BRCA* status.

Taken together, consistent evidence currently support the addition of letrozole to ovarian stimulation protocols in breast cancer patients. Not only that it reduces transient estradiol peaks, which could be somehow of concern in women with oestrogen receptor positive disease, but also increases ovarian androgen concentrations and improves response to ovarian stimulation [17].

However, in a setup of GnRH-antagonist protocol for controlled ovarian stimulation combined with the use of letrozole, a lower number of collected oocytes (7.9 versus 11.3; *p* = 0.025) and higher poor response rate (33.3% versus 3.3%; *p* = 0.014) was observed in the *BRCA*-positive cohort. The low performance of controlled ovarian stimulation was mainly observed in *BRCA*1-mutated patients [18]. However in this particular study, one could criticise the small patient population included. This contradicts a smaller yet more recent study showing no difference in 20 *BRCA* patients as compared to 36 *BRCA*-negative patients in terms of oocyte yield (13.75 versus 14.75), low response rates (8.06% versus 6.45%), number of zygotes, fertilisation rates and conception rates [19].

On the other hand, when tamoxifen was used as part of controlled ovarian stimulation, there was no difference in terms of oocytes collected (11.50 versus 11.69; *p* = 0.92) between *BRCA*-positive and *BRCA*-negative breast cancer patients, respectively [20].

### Ovarian cancer

It is estimated that about 44% of women who inherit a deleterious *BRCA*1 mutation and about 17% of women who inherit a deleterious *BRCA*2 mutation will develop ovarian cancer by the age of 80 [8]. This genetic component is also aided by the successive and continuous ovulation status of the ovary, which significantly increases the incidence of ovarian cancer [21].

Infertility itself is a risk factor for developing ovarian cancer [8]. Although based on a small number of studies, a recent Cochrane review showed a two- to four-fold increased risk of borderline tumours with fertility treatment [22].

Gronwald *et al* [23] looked at the relationship between fertility drugs and ovarian cancer in women with *BRCA* mutations. With 941 *BRCA* carriers matched to equal number of controls, there was no statistically significant association between the use of any specific types of fertility medication and the risk of ovarian cancer. The above results were also seen in patients with invasive epithelial ovarian cancer with 139 women with *BRCA*1 mutation and 33 women with *BRCA*2 mutation. Fertility treatments were not associated with cancer risk (age-adjusted OR 0.63; 95% CI, 0.38–1.05) regardless of treatment type (clomiphene citrate, OR 0.87; 95% CI, 0.46–1.63; gonadotropin use, OR 0.59; 95% CI, 0.26–1.31 as well as with IVF/ICSI, OR 1.08, 95% CI, 0.57–2.06). There seems to be no increased risk of developing breast or ovarian in patients harbouring *BRCA*1/2 mutations through controlled ovarian stimulation, yet data in the literature is lacking regarding with paucity of studies looking at controlled ovarian stimulation in patients with ovarian cancer.

Considering that ovarian cancer staging is surgical in nature, the degree of surgical intervention and removal of ovaries, fallopian tubes and the uterus is largely dependent on the stage of the disease [24]. Preservation of the uterus is generally reserved for young patients with early stage disease which desire future fertility [25]. Thus, controlled ovarian stimulation in patients with ovarian cancer must be dealt with carefully and professional input is mandatory in such a case, not only regarding its safety, but also in regards to considering the necessity of preserving the uterus or not, which entitles the patient to seek surrogacy for conception, even after freezing her own oocytes.

## Ovarian tissue cryopreservation

Ovarian tissue cryopreservation is an effective technique for fertility preservation that may be proposed to selected breast cancer patients such as those who cannot delay anti-cancer treatments or with contraindications to controlled ovarian stimulation [26].

In an already fertility compromised patient as reported in the section above, a person has to weigh both the pros and cons from ovarian tissue cryopreservation. Apart from it still being an experimental approach to fertility preservation with no strict and clear recommendations for its use, it should also be noted that there is a great deal of follicular loss in the freeze/thaw process of ovarian tissues, especially in primary follicles [27].

Following re-implantation of ovarian tissue, graft function is expected to be restored in almost all the cases at around 3–6 months, yet data on long term recovery/survival of the graft is still scarce [28]. Regarding the reproductive potential, the pregnancy rate per transplantation was estimated to be approximately 20%–30% [29], with over 130 live births reported after the transplantation of cryopreserved ovarian tissue, and almost a 100% short term ovarian function recovery after tissue re-implantation [30]. In the most recent retrospective analysis of two prospective studies, it was shown that patients who underwent ovarian tissue cryopreservation (*N* = 72), women in the *BRCA*-positive cohort tended to have a lower number of oocytes per fragment (0.08 versus 0.14; *p* = 0.193) and per square millimetre (0.33 versus 0.78; *p* = 0.153) than those in the *BRCA*-negative cohort [31].

## Attitudes towards pre-implantation genetic testing (PGT)

Patients with hereditary breast and ovarian cancer seek fertility preservation procedures in order to preserve their childbearing capacity before any prophylactic bilateral salpingo-oophorectomy surgery. Yet, patients lack counselling about the risk of transmission of the mutation to offspring with lack of knowledge and perception of the existence of PGT [32]. A systematic review including 13 studies showed that 65% to 80% of carriers lack any awareness regarding PGT for hereditary caners. If they had any prior information about PGT, the majority of participants would agree to have it as an acceptable option [33. Yet, the decision to choose between PGT and prenatal diagnosis for couples is challenging. A qualitative study including 18 couples was conducted to assess the reasons to choose PGT among well-informed couples with *BRCA*1/2 mutations. Couples choosing PGT were more concerned about medically protecting their child compared to the disadvantages related to pursuing IVF [34].

The decision making of *BRCA* carrier to undergo fertility preservation is based on their knowledge about the different oncofertility services, the safety of controlled ovarian stimulation and a thorough planning of the best fertility option before any prophylactic surgery as it is bound with a severe psychological and emotional impact.

## Gonadotropin-releasing hormone (GnRHa) suppression

In the adult ovary, >90% of the ovarian reserve is made up of primordial follicles in the resting stage prophase I. Growth initiation of follicles starts as a FSH-independent process and gonadotropin dependent growth won’t occur until later on (antral phase) [35]. As profound ovarian suppression may take several weeks to be achieved, it is unlikely that sufficient lowering of gonadotropins will be achieved within the short time available before the initiation of chemotherapy. Also, if GnRH analogues are given during the follicular phase of the cycle, they may actually cause a flare effect and create the opposite of the desired impact and defeat the purpose of actually suppressing ovarian function during chemotherapy treatment.

This raised many questions regarding how GnRH analogues actually work in fertility preservation and yet the answers in the literature remain unclear [36].

A proposed intervention in mice showed that gonadotropins enhance caspase-3 and caspase-7 and apoptosis in the theca-interstitial cells of rat pre-ovulatory follicles in culture. The elevations in caspase-3 and caspase-7 activities in theca-interstitial cells were accompanied by an increase in apoptosis [37]. Thus pituitary desensitisation, induced by GnRH agonist administration, prevents the secretion of growth factors by the FSH-dependent follicles; thus, secondarily preserving more primordial follicles in the ‘dormant’ stage, and minimising their unidirectional maturation and ultimate destruction by alkylating agents.

Moreover, it has been shown that human gonads contain independent GnRH receptors. GnRH-I and GnRH-II receptor activation may result in decreased apoptosis. Whether the GnRH agonist effect is direct on the oocyte–cumulus complex or on the granulosa cells themselves, this topic in particular requires further assessment [38].

Another possibility is that GnRH agonists may upregulate an intragonadal antiapoptotic molecule such as sphingosine-1-phosphate which serve as molecules enhanced when triggered by chemotherapy thus leading to oocyte apoptosis [39].

### Clinical data with GnRHa

Data have been promising in terms of resumption of menses up to 1–2 years after chemotherapy upon the use of GnRH agonist ovarian suppression in patients at risk of developing premature ovarian failure [40].

Lambertini *et al* [41] studied 873 patients, in an individual patient based meta-analysis from five trials. Four hundred thirty-six were randomly assigned to the GnRHa group and 437 to the control group, and where chemotherapy-induced premature ovarian failure was the primary end point in all trials. In the GnRHa group, 51 (14.1%) of 363 patients developed premature ovarian insufficiency (POI), as compared with 111 (30.9%) of 359 in the control group (adjusted OR, 0.38; 95% CI, 0.26–0.57; *p* < 0.001) without any heterogeneity. The multivariate analysis showed that only treatment with GnRHa (adjusted OR, 0.38; 95% CI, 0.26–0.57; *p* < 0.001) and younger age at diagnosis (<40 years) (adjusted OR, 0.35; 95% CI, 0.24–0.52; *p* < 0.001) were significantly associated with a reduced risk of developing chemotherapy-induced POI [41].

Del Maestro *et al* [42] demonstrated that the use of triptorelin-induced temporary ovarian suppression during chemotherapy in premenopausal patients with early-stage breast cancer reduced the occurrence of chemotherapy-induced early menopause with the launch of the PROMISE/GIM-5 trial in 16 Italian centres. The study group looked at the incidence of early menopause in young (aged 18–45 years) patients with breast cancer undergoing adjuvant or neoadjuvant chemotherapy in a randomised controlled trial. Patients received Triptorelin intramuscularly at a dose of 3.75 mg at least 1 week before the start of chemotherapy and then every 4 weeks for the duration of chemotherapy. Early menopause was defined as no resumption of menstrual activity and postmenopausal levels of FSH and estradiol 1 year after the last cycle of chemotherapy. The study included hormone receptor positive as well as hormone receptor negative breast cancer patients where hormone receptor positive patients received adjuvant treatment with Tamoxifen for up to 5 years. There was no significant difference between the chemotherapy regimens their patients received, either CMF-based (cyclophosphamide, methotrexate and fluorouracil) Anthracycline-based or Anthracycline-Taxane based treatment. With 133 patients randomised to chemotherapy alone and 148 patients randomised to chemotherapy plus triptorelin, the rate of early menopause was 25.9% in the chemotherapy-alone group and 8.9% in the chemotherapy plus triptorelin group (*p* = 0.001), thus an attributable risk reduction of 17% (95% confidence interval, −26% to −7.9%; *p* < 0.001). In a multivariate analysis, only the treatment with triptorelin was associated with a significant reduction of the risk of developing early menopause with an odds ratio for treatment-related early menopause of 0.28 (95% confidence interval, 0.14–0.59; *p* < 0.001) [42].

The PROMISE trial reported its final results in 2015 [43] with a conclusion that regardless of hormone-receptor status, premenopausal women do actually benefit from the administration of triptorelin along with chemotherapy specifically in terms of higher long-term probability of ovarian function recovery, however without a statistically significant difference in pregnancy rate. A total of 281 women were followed for a median of 7.3 years. In that period of time, the incidence of menstrual resumption at 5 years was 72.6% (95% CI, 65.7%–80.3%) among the 148 patients in the GnRHa group and 64.0% (95% CI, 56.2%–72.8%) among the 133 patients in the control group (hazard ratio (HR), 1.28 (95% CI, 0.98–1.68); *p* = 0.07).

In a more recent phase 3 trial, Moore *et al* [44] looked at a specific patient population of stage I to IIIA estrogen-receptor–negative and progesterone-receptor–negative breast cancer only and randomised them into receiving chemotherapy alone versus chemotherapy and Goserelin at a dose of 3.6 mg SC every 4 weeks beginning at 1 week before the initial chemotherapy dose and continued to within 2 weeks before or after the final chemotherapy dose. 135 with complete primary end-point data (ovarian failure at 2 years), the ovarian failure rate was 8% in the Goserelin group and 22% in the chemotherapy-alone group (odds ratio, 0.30; 95% confidence interval (CI), 0.09–0.97; *p* = 0.04) [44]. In the authors’ final analysis after a median follow up time of 5.1 years, results showed that patients in the chemotherapy + GnRHa arm had a 2.34 higher odds of at least one pregnancy versus the chemotherapy arm (23.1%, versus 12.2%, *p* = 0.03). Importantly, there was no detrimental effect on survival with the addition of GnRHa to chemotherapy.

Thus, in our view, the administration of GnRH analogues in patients receiving chemotherapy may offer a more accessible option for breast cancer patients and can be used in conjunction with traditional fertility-preservation techniques. It also offers feasibility in regard to cost, timing issues and the need for a partner yet with sometimes cumbersome side effects, such as vasomotor symptoms, mood changes, as well as potential osteoporosis. However, ovarian stimulation for embryo or oocyte cryopreservation remains the standard options to be considered even in *BRCA* patients. To date, we still lack evidence on the efficacy GnRHa in patients harbouring *BRCA* mutation. As the latter appear to have inherent ovarian function insufficiency, it remains important to understand the potential value of these agents in such patient population.

Although the updated American Society of Clinical Oncology practice guidelines state that there is conflicting evidence to recommend gonadotrophin-releasing hormone agonists (GnRHa) for medical fertility preservation, its administration is now considered as ‘standard of care’ in breast cancer patients before and during the receipt of cytotoxic agents [45] yet with limited evidence available for its use in *BRCA* patients. Importantly, GnRHa should be started preferably at least one week before the initiation of chemotherapy and continued until after the last dose of chemotherapy. As for the POEMS trial that included patients with stage I-IIIA oestrogen and progesterone negative breast cancer, patients receiving GnRH agonist in addition to chemotherapy were more likely to experience a pregnancy with a 23.1% 5-year cumulative incidence compared to only 12.2% in chemotherapy alone (*p* = 0.03) [46].

## Conclusion

The authors acknowledge that there is limited evidence on the reproductive potential and performance of fertility preservation strategies in *BRCA*-mutated breast cancer patient currently exist in the literature.

Controlled ovarian stimulation seems to be safe and does not increase the baseline risk of neither ovarian nor breast cancer in patients harbouring *BRCA*1/2 mutations, especially when utilising stimulation protocols which uses letrozole to maintain physiological levels of oestrogen.

There has been a demonstrated efficacy and safety of temporary ovarian suppression with GnRHa during chemotherapy as an available option to reduce the likelihood of chemotherapy-induced premature ovarian failure in patients harbouring *BRCA*1/2 and is endorsed widely among societies and centres around the world.

Although still experimental, one should acknowledge that there is a great deal of follicular loss in the freeze/thaw process of ovarian tissues yet with reported positive results and livebirths [28].

Prior to initiating potentially gonadotoxic therapy, physicians should discuss the risk of treatment-induced infertility and possible interventions to preserve fertility. This discussion should occur soon after diagnosis since some interventions to preserve fertility take time and could delay the start of treatment. This is where further studies are needed to deeply look on the impact of cancer on the quality of life and to enhance not only the reproductive outcome, but also the psychological outcomes including acceptance, recognition, determination, strength and inner healing. A sound and healthy psychologic status, supported by perceptive honest counselling from recent evidence, empowers the woman to strongly fight cancer.

## Conflicts of interest

All authors state that this manuscript is an original work that has not been previously published in any other peer-reviewed journal, and is not currently in press or under consideration by any other journal. All authors have neither conflicts of interest to disclose nor any financial and personal relationships with other people or organisations that could influence this work.

## Funding declaration

No funding of any kind has been provided for this manuscript.

## Authors’ contributions

Guarantor of integrity of the entire study: Suleiman Ghunaim, Ghina Ghazeeri, Hatem Azim Jr. Literature research: Suleiman Ghunaim, Dalia Khalife. Manuscript preparation: Suleiman Ghunaim, Hatem Azim Jr., Dalia Khalife. Manuscript editing: Hatem Azim Jr., Ghina Ghazeeri, Suleiman Ghunaim, Dalia Khalife.

## Figures and Tables

**Tabl 1.** Studies investigating ovarian reserve in women harbouring *BRCA* mutation.

References	Objective	Main Findings	Limitations
Oktay *et al* [18]	Oocyte yield after ovarian stimulation	Compared with controls, *BRCA*1 mutation-positive women produced lower numbers of oocytes (7.4 (95% CI, 3.1–17.7) versus 12.4 (95% CI, 10.8–14.2); *p* = 0.025) and are therefore at risk for occult POI.	Small sample size *BRCA*1 (*n* = 8) carriers, *BRCA*2 (*n* = 4) carriers (mean age: 33.1 year); *BRCA* negative−(*n* = 33; mean age: 32.8 year)
Titus *et al* [6]	Degree of DNA break due to *BRCA* mutation effect on ovarian reserve in both mice and humans	In humans, *BRCA*1 gene expression showed a significant age-related decline (*r* = 0.60; *p* < 0.001) after the age of 36 years. -*BRCA*1 mice (*N* = 3 mice/group) produced fewer oocytes in response to ovarian stimulation compared with wild-type mice (14 ± 7.8 versus 33.3 ± 0.9; *p* < 0.05) -*BRCA*2+/Δ27 and *BRCA*2Δ27/Δ27 mice had similar oocyte yields) as wild-type mice. In humans, (*N* = 60/group, mean age 36.3 ± 3.5 years) 15 patients with *BRCA*1 mutation had a mean AMH concentrations = 1.12 ± 0.73 ng/mL, *p* < 0.0001. *BRCA*2-only mutations did not have a significantly lower AMH (*N* = 9/group, AMH = 1.39 ± 1.20, *p* = 0.127)	Small population size
Lambertini *et al* [31]﻿	﻿Baseline AMH and performance of cryopreservation strategies were compared between patients with or without deleterious *BRCA* mutation.	Median AMH levels were 1.8 μg/L (IQR 1.0–2.7) and 2.6 μg/L (IQR 1.5–4.1) in the *BRCA*-positive and *BRCA*-negative cohorts, respectively (*p* = 0.109). Among patients who underwent oocyte cryopreservation (*N* = 29), women in the *BRCA*-positive cohort tended to retrieve (6.5 versus 9; *p* = 0.145) and to cryopreserve (3.5 versus 6; P = 0.121) less oocytes than those in the *BRCA*-negative cohort.	Retrospective analysis conducted in a relatively small population. Clinical deleterious effect of *BRCA* mutation on ovarian reserve, yet did not reach statistical significance
Turan *et al* [16]	Both Impact of Letrozole and *BRCA* Mutations on Fertility Preservation Cycle Outcomes	The mean number of total (15.6 (7.9) versus 10.2 (7.8); *p* = 0.004) and mature oocytes (10.4 (5.1) versus 7.8 (3.5); *p* = 0.044) and embryos frozen (7.7 (5.3) versus 5.3 (2.7); *p* = 0.043) were significantly higher after Letrozole addition to stimulation. Women with *BRCA* mutations produced significantly fewer oocytes (11.0 (8.0) versus 16.4 (7.7), *p* = 0.015) and embryos (5.1 (4.4) versus 8.2 (4.7), *p* = 0.013), compared to patients who were mutation negative even with the utilisation of Letrozole.	Secondary analysis of a prospective cohort. rFSH without Letrozole was tested in hematological cancer patients and not breast cancer patients.
Son *et al* [45]	Association between *BRCA* mutation status and serum AMH level in young, reproductive-aged patients with breast cancer	Significantly lower median AMH in *BRCA* carriers compared to controls (2.60 vs. 3.85 ng/mL, 32% reduction, *p* = 0.004) No difference in AMH level between *BRCA*1/2 mutations.	Retrospective nature, only AMH to assess ovarian reserve, no report on long term fertility.

Abbreviations: AMH: anti-müllerian hormone, BMI: body mass index, CI: confidence interval, POI: Premature ovarian insufficiency.
